# The Protein Composition and In Vitro Digestive Characteristics of Animal- versus Plant-Based Infant Nutritional Products

**DOI:** 10.3390/foods12071469

**Published:** 2023-03-30

**Authors:** Margaret E. Byrne, Elena Arranz, Francesca Bot, Laura G. Gómez-Mascaraque, John T. Tobin, James A. O’Mahony, Tom F. O’Callaghan

**Affiliations:** 1 School of Food and Nutritional Sciences, University College Cork, T12 TP07 Cork, Ireland; 2 Food Chemistry and Technology Department, Teagasc Food Research Centre, Moorepark, Fermoy Co., P61 C996 Cork, Ireland

**Keywords:** infant formula, dairy, plant protein, digestion, functionality

## Abstract

The protein composition and digestive characteristics of four commercially available infant formulae (IF) manufactured using bovine (B-IF), caprine (C-IF), soy (S-IF), and rice (R-IF) as a protein source were examined in this study. Plant-based formulae had significantly higher crude protein and non-protein nitrogen (NPN) concentrations. Static in vitro gastrointestinal digestion of these formulae, and subsequent analysis of their digestates, revealed significantly higher proteolysis of B-IF at the end of gastrointestinal digestion compared to the other formulae, as indicated by the significantly higher concentration of free amine groups. Furthermore, differences in structure formation during the gastric phase of digestion were observed, with formation of a more continuous, firmer coagulum by C-IF, while R-IF demonstrated no curd formation likely due to the extensive hydrolysis of these proteins during manufacture. Differences in digestive characteristics between formulae manufactured from these different protein sources may influence the bio-accessibility and bioavailability of nutrients, warranting additional study.

## 1. Introduction

Breastmilk is considered the most suitable form of nutrition for infants, as recommended by the World Health Organisation. However, in circumstances where breastfeeding is not possible or is discontinued, infant formulae (IF) is a suitable alternative to breastmilk to fulfil the infant’s nutritional requirements until such time that complementary foods may be introduced. Protein intake is extremely important for infants, influencing their growth, body composition, appetite, and hormonal regulation [1]. Ingredients of dairy origin typically provide the protein source for IF [2,3]. However, dairy-based formulae, such as those manufactured from bovine or caprine milk, are not suitable for all infants. For example, these formulae are unsuitable for infants diagnosed with milk protein allergy, galactosemia, or primary lactase deficiency [4,5]. Furthermore, increasing awareness among consumers regarding welfare issues and the environmental impact of animal-based foods has contributed to a purchasing preference among certain demographics for plant- over dairy-based IF in some instances [6]. Commercially available plant-based IF are typically manufactured from either soy protein isolates or rice protein hydrolysates, although other plant-based sources are currently receiving attention [7,8]. However, these protein sources vary in terms of protein quality, which is defined by (1) the ability of the protein to meet the essential and non-essential amino acid requirements of the infant, (2) its digestibility, and (3) the subsequent bioavailability of individual amino acids [9].

Protein digestibility is affected by structure formation during digestion, which impacts nutrient bio-accessibility within the gastrointestinal tract (GIT) [10,11,12]. Several research groups have studied the digestibility of IF to obtain insights into structure formation and degradation during digestion. To date, the primary research focus in this area has been on dairy-based formulae and their protein fractions, where differences in the characteristics of the gastric curd formed during digestion of bovine IF, caprine IF and their ingredients have been reported [13,14,15,16,17,18]. Comparatively few studies have examined the digestibility of IF manufactured from plant protein sources. Nguyen et al. [19] reported reduced digestibility of a model infant formula manufactured from soy protein compared to formulae manufactured from bovine milk protein. Le Roux et al. [8] reported comparable digestibility between a reference IF made from bovine milk protein and formulae in which bovine milk protein was partially substituted with faba bean or pea protein, while formulae in which the bovine milk protein was partially substituted with rice or potato protein showed impaired digestibility. In addition, Alonso-Miravalles et al. [7] reported similar digestibility of an IF manufactured from lentil protein compared to a commercially available soy protein IF. The quality and digestibility of protein is also an important consideration in relation to the release of bioactive peptides during digestion which could have antihypertensive, hypocholesterolemic, immunomodulatory, antioxidant, antimicrobial, anti-diabetic, opiate, and hepatoprotective effects [20,21].

A variety of different models and experimental conditions have been used to replicate infant digestion across these studies, with slight but important variations in physiological parameters, such as digestion time, pH, and enzyme activities. To overcome this, a standardised in vitro static digestion method based on an extensive review of current knowledge on infant digestion physiology [22] and aligned with the INFOGEST standardised static in vitro digestion method replicating adult digestion [23,24] was proposed by Ménard et al. [25], thereby improving the reproducibility and aiding in the comparison of results obtained from different studies.

In this context, the objectives of the current study were to analyse the protein composition and profile of commercially available infant formulae manufactured from animal- and plant-sourced ingredients and to study their digestive characteristics *in vitro*.

## 2. Materials and Methods

### 2.1. Infant Formulae Sample Collection and Preparation

Four commercially available IF powders suitable for infants aged 0–6 months were selected: bovine milk-based IF (B-IF), caprine milk-based IF (C-IF), soy-based IF (S-IF), and rice-based IF (R-IF). All the samples were purchased in a local supermarket in Ireland except the rice-based formula, which was purchased from a supermarket in France. Reconstituted IF samples were prepared in ultra-pure water according to the manufacturer’s recommendations.

### 2.2. Nitrogen Fraction and Protein Analysis of Infant Formulae

The total nitrogen content of the reconstituted IF samples was determined using the Kjeldahl method [26] with 3 mL aliquots of the reconstituted formula. The crude protein content was calculated using a nitrogen-to-protein conversion factor of 6.25 for B-IF, C-IF, and S-IF, while 5.95 was used for R-IF. The non-protein nitrogen (NPN) concentration was determined by measuring the nitrogen content of the 12% trichloroacetic acid (TCA) soluble fraction of each formula using the Kjeldahl method. True protein was calculated from the results for total nitrogen (N) and non-protein nitrogen (NPN) as follows:

True Protein (%) = (N-NPN) × nitrogen to protein conversion factor

Reversed-phase high-performance liquid chromatography was performed, as reported by Vogelsang-O’Dwyer et al. [27], to obtain further details on the peptide profiles of the 12% TCA soluble fractions of the infant formulae. Only minor changes were made to the final conditions between samples to have better equilibration conditions (Table S1). Reconstituted infant formulae were first defatted by centrifugation (15,000× *g* at 4 °C) for 1 h. TCA (24%) was then added to the supernatant at a ratio of 1:1 to achieve a final TCA concentration of 12%, after which the samples were filtered using 0.45 µm syringe filters (Minisart^®^ RC25, Göttingen, Sartorius AG, Göttingen, Germany) and transferred to glass vials. Samples were analysed by reversed-phase high-performance liquid chromatography (RP-HPLC) (Agilent 1220 Infinity II LC, Santa Clara, CA, USA) equipped with an Aeris Widepore XB-C-18 column (3.6 µm particle size, 250 × 4.6 mm: Phenomenex, Cheshire, UK). Gradient elution was carried out with a mixture of solvents containing 0.1% TFA in ultrapure water (solvent A) and 0.1% TFA in acetonitrile (ACN) (solvent B). Separations were performed using the program reported in Supplementary Table S1. Before injecting samples, the column was pre-equilibrated under the starting conditions for 10 min at a flow rate of 0.75 mL/min, column temperature 45 °C, and detection at 214 nm. The sample injection volume was 40 μL.

### 2.3. Static In Vitro Digestion

Static in vitro digestion of each IF was carried out using the method of Ménard et al. [25], with minor modifications. Briefly, digestion was performed in an incubator at 37 °C, with agitation provided by a rotating wheel set to 15 rpm. The gastric phase of digestion was performed using porcine pepsin (Sigma, Burlington, MA, USA, P6887) to provide a pepsin activity of 268 U/mL of gastric content. The gastric phase of digestion was carried out at pH 5.3 and was 60 min in duration. For the intestinal phase, the pH of the gastric chyme was increased to pH 6.6, at which point porcine pancreatin (Sigma, P7545) and bovine bile extract (Sigma, B8631) were added to provide a trypsin activity of 16 U/mL and a concentration of 3.1 mM of intestinal content, respectively. CaCl_2_ was added separately, at a concentration of 3 mM, to the intestinal fluid. The intestinal phase was halted by the addition of 50 µL of 0.1 M Pefabloc^®^ SC (Sigma, 76307), providing time point I60. Samples at the gastric timepoints G0, G30, and G60 were obtained by adjusting the pH of the gastric chyme to pH 8 using 1 M NaOH immediately after its initiation, 30 min, and 60 min, respectively, and then adjusted to 5.3 for further analysis. Digestion was carried out in triplicate for each IF and control with distilled water. Samples collected at each time point were divided into Eppendorf tubes in 1 mL aliquots, blast frozen at −20 °C for 20 min (Zanussi, Pordenone, Italy), and stored at −80 °C until further analysis.

### 2.4. Sodium Dodecyl Sulphate-Polyacrylamide Gel Electrophoresis

The protein profile of the reconstituted IF and IF digestates were analysed using sodium dodecyl sulphate-polyacrylamide gel electrophoresis (SDS-PAGE) using pre-cast Mini-Protean Tetra cell TGX 4–20% acrylamide gels (Bio-Rad Laboratories Inc. Hercules, CA, USA) under reducing conditions with β-mercaptoethanol as the reducing agent. Samples were centrifuged at 1000 rpm for 30 min. Aliquots of sample providing a protein load of 10 µg were loaded into the wells of the gels, and electrophoretic separation was performed at a constant voltage of 160 V. The gels were stained using Coomassie blue R250 and de-stained using a solution of water, methanol, and acetic acid at a ratio of 50:40:10, respectively. The stained SDS-PAGE gels were scanned using a desktop flatbed scanner (HP Scanjet G4010, HP, Leixlip, Ireland).

### 2.5. Degree of Hydrolysis

The degree of protein hydrolysis in reconstituted formulae and digestates was assessed using an O-phthaldialdehyde (OPA) spectrophotometric assay. Reconstituted IF and digested samples were pre-treated with TCA (final TCA concentration 3.12%) and centrifuged at 10,000× *g* for 30 min at 22 °C. The supernatant was filtered using 0.45 µm syringe filters (Minisart^®^ RC25, Göttingen, Sartorius AG, Göttingen, Germany). The OPA reagent used was as described by Nielsen et al. [28]. L-leucine solutions prepared in phosphate-buffered saline at concentrations of 0–10 mM were used to create a calibration curve. L-leucine solution/sample (50 µL) and 1 mL of OPA reagent were placed into Eppendorf tubes. The reaction was allowed to proceed for 15 min, at which time the absorbance was measured at 340 nm in a 1 mL quartz cuvette using a Cary 300 Bio UV-visible spectrophotometer (Varian Inc., Palo Alto, CA, USA.). Results were corrected with control digestion using distilled water.

### 2.6. Amino Acid Analysis

The free amino acid (FAA) composition of the IF digestates at the end of gastrointestinal digestion (I60 samples) was analysed using cation exchange liquid chromatography coupled with post column ninhydrin detection [29]. Deproteinisation of I60 samples for FAA analysis was achieved by mixing equal volumes of 24% TCA and I60 sample and allowing to rest for 10 min, after which the sample was centrifuged at 14,000 rpm for 10 min to separate the supernatant. The resulting samples were diluted 1 in 2 with the internal standard, norleucine to give a final concentration of 125 nm/mL. Amino acids were quantified using a Jeol JLC-500/V amino acid analyser (Jeol Ltd., Garden city, UK) fitted with a Jeol Na+ high-performance cation-exchange column. FAA analysis of the intestinal digestates was performed in triplicate, at the Food Chemistry & Technology Department (Teagasc Food Research Centre, Moorepark, Fermoy, Cork,, Ireland). Results were corrected with control digestion with distilled water.

### 2.7. Microstructural Analysis

The microstructural properties of the IFs before, during, and after digestion were assessed using confocal laser scanning microscopy (CLSM). Aliquots (400 μL) of the reconstituted formula or digestates were stained with 10 μL of 0.1% Fast Green FCF (aq.) and 20 μL of 0.02% Nile Red (in 1,2-propanediol), after which samples were transferred to microscopy slides and covered with glass coverslips. A Leica TCS SP5 confocal laser scanning microscope (Leica Microsystems CMS GmbH, Wetzlar, Germany) was used to observe the stained samples using a 63×/1.4 oil immersion objective. Fast green FCF was excited at 633 nm using a He/Ne laser, and the corresponding emission filter was set at 685–750 nm. Nile red was excited at 488 nm using a diode-pumped solid-state laser, and the corresponding emission filter was set at 600–615 nm. The analysis was performed at the Teagasc National Food Imaging Centre (Moorepark, Cork, Ireland).

### 2.8. Simulated acid Coagulation Properties of Infant Formulae

The coagulation properties of IFs under acidic conditions were determined using a method previously described by O’Callaghan et al. [30], with modifications accounting for the use of infant formula as the starting material. Aliquots (25 mL) of IF were pre-warmed to 30 °C in a water bath. Glucono-δ-lactone (GDL; 2%, *w*/*v*) was added to the samples and stirred vigorously for 30 s. Small amplitude oscillatory rheological measurements were carried out using a Discovery Hybrid 2 Rheometer (TA instruments, Crawley, West Sussex, UK) fitted with a concentric cylinder geometry at 30 °C. A time sweep analysis was performed using the following conditions: 5 s temperature equilibration at 30 °C, 15 s pre-shear at 50 s^−^^1^, and 10 s equilibration, followed by oscillation at 0.1% strain at a frequency of 1 Hz. A second treated IF sample remained in the adjacent water bath at 30 °C, the pH of which was monitored continuously until pH 4.6 was reached, at which point the time sweep was stopped. The gel point was defined as the point at which the value for the storage modulus (Gʹ) was >1 Pa.

### 2.9. Statistical Data Analysis

Statistical analysis was performed using IBM SPSS Statistics V28 (IBM Statistics Inc., Armonk, NY, USA). Statistical differences between the means of experimental data from N analysis, FAA composition, and the acid coagulation properties of IF were assessed by analysis of variance (ANOVA), followed by Tukey’s posthoc analysis. Analysis of the OPA assay results was carried out using repeated measures ANOVA with posthoc Bonferroni adjustment. Results were considered statistically significant when *p* < 0.05. Multivariate statistical analysis was carried out on the results of the free amino acid analysis using MetaboAnalyst software (www.metaboanalyst.ca, accessed on 7 February 2023), from which Figure S1 was generated.

## 3. Results and Discussion

### 3.1. Protein and Amino Acid Composition of Formulae

The nitrogen and crude protein content of the plant-based formulae was found to be significantly higher than the dairy-based formulae (Table 1). Plant proteins are known to be deficient in certain essential amino acids; for example, methionine is the most limiting amino acid in soy protein, while rice protein has been reported to contain limited amounts of lysine [4,31,32]. In addition, plant proteins have been reported to have a lower digestibility than milk proteins [4,32]. To compensate for these differences, a higher minimum protein concentration is recommended for formulae manufactured from plant protein [33,34], as reflected in the results from this study.

The NPN fraction is diverse in terms of nitrogenous compounds, which remain soluble in 12% TCA [35]. While the approach described in Section 2.2 of this study is an international standard method to determine the concentration of NPN in milk, TCA precipitation-based methods have previously been utilised in the measurement of NPN both in soy IF [36] and other plant protein-based IF [37,38]. However, there is still limited information available on this in the literature, and comparable results for the concentration of NPN in hydrolysed rice protein formula are not readily available in the literature.

The concentration of NPN was significantly different across the different formulae, and R-IF > S-IF > C-IF > B-IF when expressed as a % of total N (Table 1), which is in line with results presented by Prosser et al. [39]. This is also in agreement with previous literature outlining the concentration of NPN in bovine and caprine milk, with values reported of 5–6% of milk nitrogen as NPN [35] and 8.1% of milk nitrogen as NPN [39] for bovine and caprine milk, respectively.

The concentration of NPN in S-IF was higher than that in B-IF and C-IF at 0.07%, making up 24.6% of the total nitrogen content in these formulae. This is in agreement with findings by Donovan and Lonnerdal [36], who reported the concentration of NPN to be 0.14–0.34 g/L in bovine IF and 0.12–0.85 g/L in soy IF. Similarly, the concentration of NPN in soy protein isolate has previously been reported to be higher than that of bovine milk at 18.3% of total nitrogen, with variation in this concentration depending on the soy cultivar and protein extraction method used [37].

**Figure 1:**
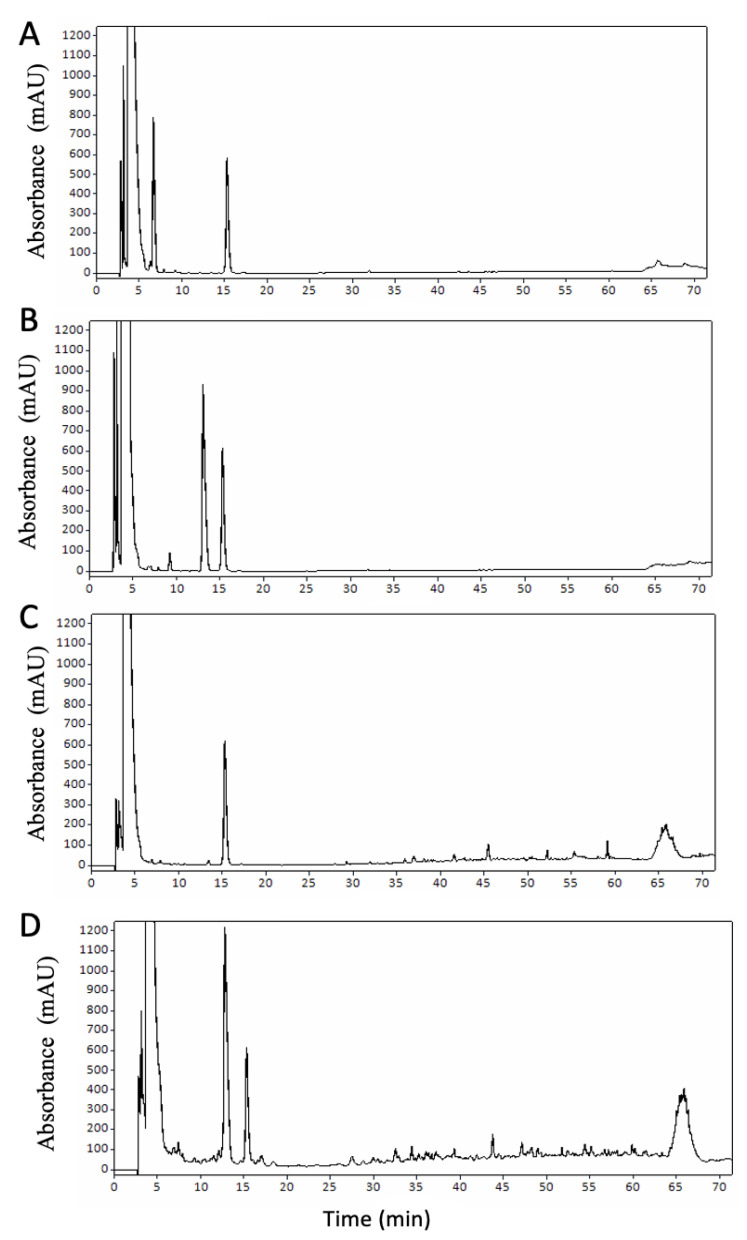
RP-HPLC chromatograms of 12% TCA soluble fraction of infant formula (IF) samples; (**A**) B-IF (bovine IF), (**B**) C-IF (caprine IF), (**C**) S-IF (soy IF), (**D**) R-IF (rice IF).

The concentration of NPN was considerably higher for the rice protein formulae at 0.21% for R-IF, corresponding to over 86% of the total nitrogen content in this formula (Table 1). This may be attributable to the extensive hydrolysis of the rice proteins in this formula, with rice protein hydrolysate ingredients identified in the ingredient declaration, resulting in the presence of small peptides and free amino acids, which would be soluble in 12% TCA. Chromatograms obtained from RP-HPLC analysis of the 12% TCA-soluble fraction of each formula (Figure 1A–D) also provide evidence for the presence of small peptides within this fraction of the plant protein formulae. Figure 1 demonstrates a significantly larger number of minor peaks with retention times in the range of 16–65 min present in the chromatograms for S-IF, and particularly R-IF, compared to B-IF and C-IF. In each of the samples, major peaks at ~5 min which represents the injection peak and most hydrophilic compounds, and peaks at ~15 min resulting from TCA, were observed. A previous study of rice protein-based infant formulae reported that 96–100% of the peptides in these formulae had a molecular weight of <5 kDa [40].

The generally poor solubility of plant proteins [41,42], and in particular rice [43] and soy [44] proteins, is widely recognised as one of their functional limitations. Hydrolysis of these proteins has been demonstrated to significantly improve protein solubility, thereby enhancing their functionality for use as an ingredient in IF [32,45].

### 3.2. Protein Profile during Digestion

**Figure 2:**
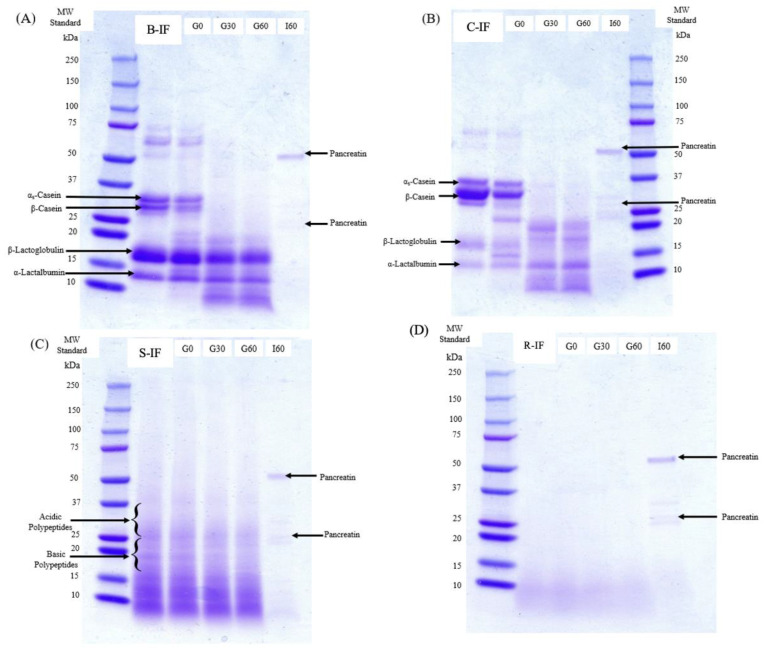
SDS-PAGE electrophoretograms of infant milk formula (IMF) samples displaying reconstituted (undigested) and digested formulae at various gastric (G0, G30, G60) and intestinal (I60) time points. (**A**) B-IF (bovine IF), (**B**) C-IF (caprine IF), (**C**) S-IF (soy IF), and (**D**) R-IF (rice IF).

Figure 2A,B show the electrophoretograms of B-IF and C-IF, respectively. The bands between 25 and 37 kDa representing the caseins were considerably more intense for C-IF than for B-IF, particularly the band representing β-casein, demonstrating a higher casein-to-whey ratio in C-IF than B-IF. This was also shown by the presence of whole goat milk and goat milk powder in the ingredient declaration in C-IF, while in B-IF, the casein-to-whey ratio has been altered by combining whey protein and skimmed milk, more closely reflecting that of human milk. Prosser [46], on review of the topic, discussed how studies have demonstrated that formula made from whole goat milk without adding whey can satisfy the protein and amino acid requirements of the formula and also have a similar curd strength as cow milk formula with a 60:40 whey-to-casein ratio. The review also highlights how differences exist between the casein profile of goat milk compared to bovine milk, notably with higher levels of β-casein and lower levels of ⍺_S1_-casein in goat’s milk. The band for β-lactoglobulin at 18 kDa was correspondingly larger in B-IF than in C-IF, also potentially reflecting the difference in their casein to whey ratio. Additionally, the more distinct band for β-casein in C-IF indicates dominance in this formula, as would be expected for caprine IF, in contrast to bovine milk, where α_s1_-casein is the dominant casein [47].

The proteolytic action during the gastric phase of digestion was clearly evident in the electrophoretograms of B-IF and C-IF (Figure 2A,B). After 30 min of gastric digestion, the bands representing intact caseins were negligible, with the appearance of additional, lower molecular weight bands, demonstrating the hydrolysis by pepsin of the caseins. The almost complete hydrolysis of casein in the gastric phase of digestion has similarly been reported by Ménard et al. [25], who observed residual casein of 10.9 ± 6.5% after 60 min of gastric digestion of an infant formula. Phosanam et al. [17] also observed that most of the casein in a range of model IF manufactured from bovine milk protein was hydrolysed after 60 min of gastric digestion using the same method. However, variations in the gastric digestion rate of casein were reported in studies of infant digestion using alternative static in vitro models. For example, Nguyen et al. [19] reported slower gastric digestion, whereby less than 20% of casein in model IF manufactured from bovine milk was hydrolysed after 60 min of gastric digestion. In contrast, Dupont et al. [48] reported the absence of intact β-casein after just 10 min of gastric digestion.

In contrast to the caseins, the bands representing the whey proteins remained intact throughout gastric digestion. The resistance of whey protein to in vitro gastric digestion has been widely reported [13,17,19,25,48] and is due to their compact, globular structure and their stability in the acidic environment of the stomach [49].

The electrophoretogram for S-IF (Figure 2C) displayed few distinct protein bands compared to B-IF and C-IF. Soybeans contain two primary seed storage proteins: glycinin and β-conglycinin [19,50]. The lack of protein bands at the molecular weights of the β-conglycinin subunits, α (76 kDa), α’ (72 kDa), and β (53 kDa), and the lack of clear bands for the acidic and basic subunits of glycinin suggests that the protein in the S-IF was partially hydrolysed. Electrophoretograms of the rice protein formulae (Figure 2D) and the absence of any bands >15 kDa in this formula is likely a result of the extensive protein hydrolysis in the native product pre-digestion.

### 3.3. Degree of Protein Hydrolysis

There was a significant change in free amine groups in each sample as digestion progressed from reconstituted samples through to G60 and I60 stages, as expected; however, the levels of free amine groups in R-IF remained relatively stable between G0 and G60 timepoints as is shown in Figure S2. The concentration of free amine groups of digested protein was significantly higher in R-IF than the other formulae during the gastric stage of digestion, further demonstrating the high degree of hydrolysis of these reconstituted formulae. These results are similar to those of Corrigan and Brodkorb [13], who reported that hydrolysis of dairy ingredients resulted in increased speed of gastric digestion compared to the native form.

**Figure 3:**
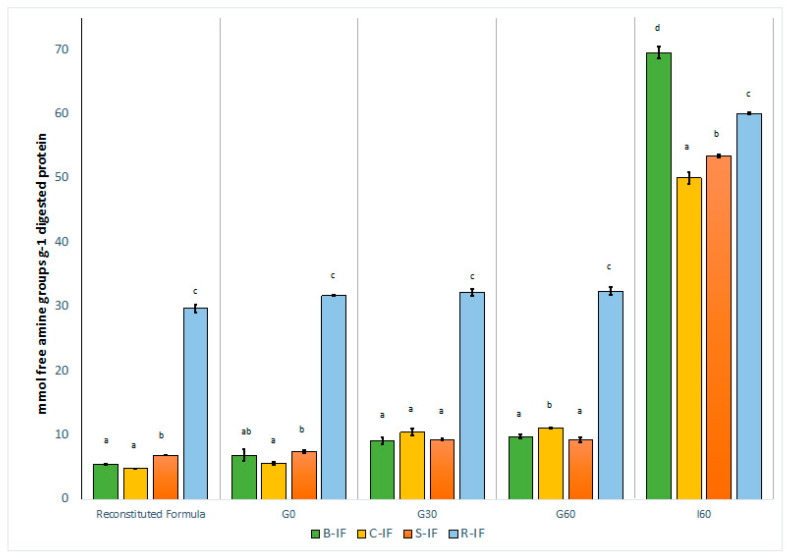
Concentration of free amine groups (mmol/g digested protein) in reconstituted (undigested) and digested infant formulae (IF) at various gastric (G0, G30, G60) and intestinal (I60) time points. B-IF (bovine IF), C-IF (caprine IF), S-IMF (soy IF), and R-IF (rice IF). Data are given as mean of three independent replicates ± standard deviation. Means not sharing superscript letters within a section represents statistical significance (*p* < 0.05).

The results shown in Figure 3 also show greater hydrolysis of proteins during the intestinal stage than during the gastric stage of digestion, agreeing with the SDS-PAGE electrophoretograms of each formula, which display an absence of peptides >10 kDa in size at I60 (Figure 2). The static, in vitro method applied in this study, was designed to replicate the digestive system of an infant at 28 d of age [25]. At this stage of development, pepsin secretion is low at 10–20% of adult levels [22,51]. Furthermore, gastric pH is much higher in infants than in adults at pH ~4–5 vs. pH 2 in the fasted state, respectively, while pepsin has optimal activity at a pH of 1.5–2 [22,51]. Regardless of the lower pepsin secretion and relatively high gastric pH, casein was fully degraded in B-IF and C-IF formulas at G60. In the intestinal stage of digestion, the pH and trypsin concentration are similar to that of adults [51]. This, combined with the preparation of protein for further digestion within the gastric stage, would lead to a greater capacity for proteolysis within the intestinal stage.

Furthermore, B-IF was found to contain a higher concentration of free amine groups than C-IF after 60 min of intestinal digestion, suggesting more extensive proteolysis of B-IF at this point. Few studies have compared the digestion of infant formulae manufactured from bovine and caprine milk protein, with most focusing on the gastric phase of digestion rather than the intestinal phase [15,18]. Zhou et al. [52], using a double-blind randomised control trial, examined the nutritional adequacy of goat milk infant formula (80:20, casein–whey profile) for term infants. This study examined goat milk without added whey proteins in comparison with a control bovine-based formula with a 60:40 whey casein profile. While there was some variation in the values of blood biomarkers and amino acids between groups, the mean biomarker values were within the normal reference range, and the nutritional outcomes in infants did not differ between groups. Using a piglet model, Rutherfurd et al. [53] examined the amino acid digestibility of goat- and cow-milk-based infant formulas and reported there was no significant difference in the nitrogen retention of piglets fed the different formulas and concluded that both formulas were similar in terms of protein quality.

Using a dynamic in vitro model replicating infant digestion, Maathuis et al. [16] reported a higher true ileal protein digestibility for a caprine IF compared to a bovine IF with the same whey-to-casein ratio (78.3% vs. 73.4%, respectively), contrasting with the results from the present study. Similarly, in a study examining the in vitro digestion of a range of milks from different species, bovine milk was found to release a significantly lower concentration of free amino groups than caprine milk at the end of intestinal digestion (*p* < 0.05) [54]. However, these studies compare IF/milk with the same casein-to-whey ratio. Therefore, the higher casein-to-whey ratio of C-IF compared to B-IF may have inhibited its intestinal digestion, as similarly observed by Phosanam et al. [17] in their investigation of the effect of whey-to-casein ratio of a cow’s milk IF on its digestion.

S-IF exhibited a lower degree of hydrolysis at I60 than B-IF (Figure 3). Similarly, Nguyen et al. [19] reported lower digestibility of a model soy IF during the duodenal phase of digestion than a model IF manufactured from bovine milk. Soy protein is known to contain protease inhibitors, such as the Kunitz trypsin inhibitor and Bowman–Birk inhibitor, which, if present, would contribute to the lower degree of proteolysis observed in the soy IF [55]. However, these inhibitors may be inactivated by appropriate technological treatments, such as heat treatments, including roasting, autoclaving, and ultra-high temperature (UHT) treatment, and should therefore have minimal effects on the digestion of soy IF [19,56]. Additionally, the reduced digestibility of S-IF compared to B-IF may arise from the unique secondary structural properties of soy protein. Carbonaro et al. [57] reported a strong, negative linear correlation coefficient (*r* = −0.98) between the proportion of β-sheets in the secondary structure of a variety of proteins and their values for digestibility. This is explained by the high degree of hydrophobic interactions within the β-sheet structure, which are associated with protein-protein interaction and the formation of protein aggregates, particularly after heat treatment, which can limit the digestibility of the soy protein [19,57].

In spite of the high degree of hydrolysis observed in the reconstituted rice protein formula before digestion, B-IF exhibited a higher degree of proteolysis than the rice protein-based formula after 60 min of intestinal digestion (Figure 3). While there are limited data available on the digestion of rice protein formulae, a study by Alonso-Miravalles et al. [7] did report that an infant formula manufactured from rice protein hydrolysates had significantly higher intestinal digestibility compared to a commercially available formula manufactured from soy protein isolate. Le Roux et al. [8] reported that a model infant formula manufactured with 50% of the protein content from unhydrolyzed rice protein concentrate and the remaining protein from bovine skim milk protein was significantly less susceptible to hydrolysis than a reference infant formula consisting entirely of bovine milk protein. Therefore, the lower degree of hydrolysis in the rice protein infant formula may be due to the remaining intact peptides in rice protein hydrolysate formulae. For example, previous studies have identified that type 1 protein bodies in rice protein, which are rich in prolamin (2–7% of the protein in milled white rice), are resistant to pancreatic proteolysis [43,45,58], therefore intact prolamin peptides, if present, may result in the decreased digestibility of the rice protein hydrolysate formulae compared to the bovine IF in this study.

### 3.4. Free Amino Acid Composition of Simulated Gastrointestinal Digests

The free amino acid (FAA) composition of the intestinal digestates (I60) is given in Table 2. In line with the higher degree of hydrolysis observed for B-IF at I60 (Figure 3), B-IF was similarly found to have significantly higher concentrations of several FAAs at this stage of digestion. There were significant differences (*p* < 0.05) in the concentration of cysteic acid between B-IF and C-IF, S-IF, and R-IF.

There was no significant difference in the concentration of cysteic acid between R-IF and S-IF. Threonine concentration was significantly higher in R-IF than in other formulas (*p* < 0.05), and there was a significant difference between B-IF and C-IF, B-IF and R-IF, C-IF and R-IF, and S-IF and R-IF. Serine concentrations of S-IF and R-IF were significantly higher than that of C-IF. Alanine concentrations were significantly higher in R-IF than that in B-IF, C-IF, and S-IF.

Valine concentration of the intestinal digestates was significantly lower in C-IF than that of B-IF and R-IF. B-IF contained significantly more free methionine than the C-IF, S-IF, and R-IF (46.5 µg/mL digesta compared to 12.30, 33.41, and 13.3 µg/mL digesta, respectively). Isoleucine concentration of the R-IF digestates was significantly higher than that of B-IF, S-IF, and C-IF (*p* < 0.05), while the leucine concentration of C-IF was significantly lower than that of B-IF and R-IF. Phenylalanine levels were significantly lower in C-IF than in other formulae (*p* < 0.05), and B-IF had significantly higher levels of lysine than C-IF and S-IF. S-IF had significantly lower tryptophan than that of C-IF and R-IF.

The arginine concentration of the plant-based formula was significantly higher than that of both B-IF and C-IF. These significant differences across the FAA profile of digestates are also evident in the principal component analysis (PCA) demonstrated in Supplementary Figure S1, where the first two principal components, which account for 79.1% of the total variance, enabled clear discrimination between the different formulae.

### 3.5. Microstructural Changes during Digestion

**Figure 4:**
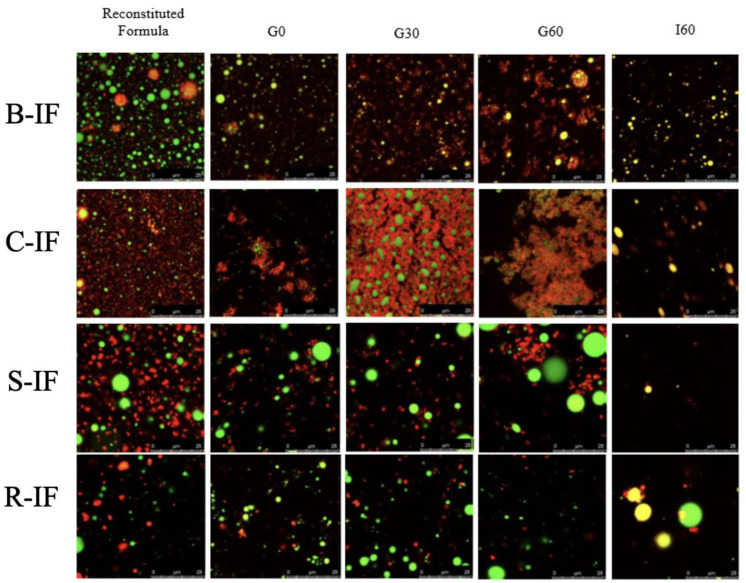
CLSM micrographs of infant milk formula (IF) samples at each digestion timepoint. B-IMF (bovine IF), C-IF (caprine IF), S-IF (soy IF), and R-IF (rice IF). Red colour represents protein, and green colour represents fat. Scale bars are 25 µm.

CLSM images for the infant formulas at each time point of digestion are presented in Figure 4. The micrographs of the reconstituted formulae before digestion showed substantial structural differences between the samples, especially between dairy and plant-based formulations, with the latter exhibiting larger protein aggregates, which could be indicative of low protein solubility. This is despite hydrolysis of the protein ingredients in these formulae, evident in Figure 2 and Figure 3.

During digestion, microstructural changes were also evident among the formula samples. The images for B-IF and C-IF show the progressive formation of aggregates and coagulation of proteins from the early stages of gastric digestion, especially for the latter, for which coagulation can be observed as early as in sample G0. This was likely due to the decrease in pH to 5.3 during the initiation of the gastric phase of digestion, which approached the isoelectric point of the caseins (pH 4.6), more abundant in C-IF than in B-IF samples. The coagulation of dairy proteins at the early stages of digestion is widely reported [59] and depends on a number of factors, including processing history and thermal treatment [60] but also the whey-to-casein ratio [61]. In contrast, no coagulation was observed in R-IF during the gastric phase, with only slight coagulation evident at the end of the gastric phase for S-IF. Structural differences during digestion may influence gastric emptying rates, with a potential impact on nutrient uptake and satiety responses [62].

### 3.6. Acid Coagulation Properties of Infant Formulae

The protein source from which infant formula is manufactured was shown to have significant effects on its acid gelation properties. The results presented in Table 3 indicate that the storage modulus (Gʹ), which is representative of the elastic or solid-like characteristics of a substance, was significantly higher for the formulae manufactured using dairy proteins than those using plant-based proteins (*p* < 0.05). Within the dairy-based formulae, C-IF reached a significantly higher complex modulus (G*) value at pH 4.6 than B-IF (6.89 Pa vs. 3.71, respectively *p* < 0.05). This is also likely to be due to the higher casein–whey ratio of C-IF, resulting in the formation of increased crosslinks between micelles leading to stronger gel networks. This was also reported by Wang et al. [63], where under acid conditions, a caprine IF with a whey–casein ratio of 20:80 had a higher value for G* compared to a bovine IF with a whey–casein ratio of 60:40. However, the authors found that a bovine IF with a whey-to-casein ratio of 20:80 had a much higher G* value than the caprine IF with the same whey–casein ratio [63]. This was attributed to the higher ratio of β-casein to α_s1_-casein and larger casein micelles in goat milk compared to cow’s milk, leading to the formation of a softer, more fragile curd when products with the same whey-to-casein ratio produced from these milks are compared [18,47,63]. The formation of large, firm aggregates of C-IF during the gastric phase of digestion may further explain its lower digestibility in comparison to the other formulae, whereby the aggregates formed may have impeded the accessibility of digestive enzymes to their substrates [17].

Similar to the lack of structure formation observed for plant protein-based formulae in the CLSM micrographs of their digestates (Figure 4), these formulae did not achieve a Gʹ value of >1 Pa under acid conditions (Table 3). This is to be expected given the extensive hydrolysis of the proteins in the native rice-based IMF pre-digestion compared to the more intact nature of the proteins in the other formulae. The impact of protein hydrolysis on gel-forming properties has previously been described for several protein sources [64] and therefore may explain the absence of acid gel formation for these formulae. Furthermore, the lower protein content of S-IF (1.73%) would be expected to contribute to its poor gel-forming capabilities compared to soy milk (2.95% protein), for example [65].

## 4. Conclusions

The results of the present study have demonstrated differences in the protein composition of dairy- and plant-based infant formulae, while there are limitations to this study given the wide variety of commercial formula and formats of ingredients available on the market today between fresh milk versus powdered ingredients, hydrolysed and unhydrolyzed ingredients sources. Nevertheless, in vitro digestion of these formulae has demonstrated that the source of protein used in the manufacture of infant formula significantly impacts its digestive properties in terms of both structure formation within the gastric phase of digestion and overall digestibility. As such, this may imply altered digestive behaviour when consumed by an infant, which could impact the bioaccessibility and bioavailability of nutrients from these formulae, and therefore, these results may have implications for the optimisation of infant formulae composition and warrant further study.

## Figures and Tables

**Table 1:** Total nitrogen (N), crude protein, non-protein nitrogen (NPN), and true protein of infant formulae (IF) samples; B-IF (bovine IF), C-IF (caprine IF), S-IF (soy IF), R-IF (rice IF). Data are presented as mean of triplicate independent replicates (% *w*/*v*) ± standard deviation.

IF	Total N (%)	Crude Protein (%)	NPN (%)	NPN as % of Total N (%)	True Protein (%)	*p*-Value *
B-IF	0.20 ± <0.01 ^c^	1.25 ± 0.01 ^c^	0.016 ± <0.01 ^d^	8.10 ± 0.20 ^d^	1.15 ± 0.01 ^b^	<0.001
C-IF	0.20 ± <0.01 ^c^	1.25 ± 0.02 ^c^	0.023 ± <0.01 ^c^	11.5 ± 0.65 ^c^	1.11 ± 0.01 ^c^	<0.001
S-IF	0.27 ± <0.01 ^a^	1.67 ± 0.01 ^a^	0.060 ± <0.01 ^b^	22.7 ± 0.69 ^b^	1.29 ± 0.02 ^a^	<0.001
R-IF	0.24 ± < 0.01 ^b^	1.44 ± 0.01 ^b^	0.210 ± <0.01 ^a^	86.7 ± 0.69 ^a^	0.19 ± 0.01 ^d^	<0.001

* *p*-value represents statistical result from one-way ANOVA analysis, with posthoc multiple comparisons indicated within the table. Means not sharing superscript letters within a column indicate statistical significance (*p* < 0.05).

**Table 2:** FAA composition of intestinal digestates (µg/mL digesta) of infant formulae (IF) samples; B-IF (bovine IF), C-IF (caprine IF), S-IF (soy IF), R-IF (rice IF). Data are presented as mean of three independent replicates ± standard deviation.

	Amino Acid	B-IF	C-IF	S-IF	R-IF	*p*-Value *
Essential Amino Acids	Histidine	141 ± 9.7	138 ± 5.3	131 ± 32.5	140 ± 8.4	0.881
	Isoleucine	59.7 ± 5.8 ^a^	35.7 ± 1.7 ^b^	59.2 ± 16.3 ^ab^	102 ± 5.8 ^c^	<0.001
	Leucine	250 ± 29.6 ^a^	122 ± 7.5 ^b^	200 ± 73.0 ^ab^	246 ± 14.7 ^a^	0.015
	Lysine	354 ± 23.7 ^a^	125 ± 9.4 ^b^	231 ± 62.1 ^b^	275 ± 13.0 ^ab^	0.009
	Methionine	46.5 ± 2.3 ^a^	12.3 ± 2.3 ^c^	33.4 ± 8.3 ^b^	13.3 ± 2.1 ^c^	<0.001
	Phenylalanine	253 ± 24.1 ^a^	105 ± 23.5 ^b^	209 ± 57.3 ^a^	196 ± 12.8 ^a^	0.004
	Threonine	72.0 ± 22.9 ^b^	29.7 ± 2.8 ^c^	53.4 ± 15.7 ^bc^	123 ± 7.3 ^a^	<0.001
	Tryptophan	127 ± 34.1 ^ab^	163 ± 18.2 ^a^	89.4 ± 31.9 ^b^	169 ± 7.9 ^a^	0.009
	Valine	105 ± 8.1 ^a^	55.8 ± 6.87 ^b^	87.8 ± 31.8 ^ab^	102 ± 4.9 ^a^	0.026
Conditionally Essential Amino Acids	Cysteine	50.3 ± 7.5	62.2 ± 4.8	49.0 ± 16.5	62.8 ± 6.8	0.249
Non-Essential Amino Acids ^1^	Cysteic Acid	51.4 ± 3.1 ^b^	71.4 ± 11.4 ^c^	15.1 ± 4.3 ^a^	12.97 ± 3.4 ^a^	<0.001
	Tyrosine	159 ± 16.8	125 ± 2.6	133 ± 52.2	184 ± 14.0	0.121
	Alanine	47.4 ± 5.2 ^a^	33.4 ± 2.2 ^a^	49.6 ± 13.5 ^a^	83.0 ± 1.8 ^b^	<0.001
	Arginine	185 ± 11.5 ^b^	156 ± 4.5 ^b^	338 ± 94.2 ^a^	345 ± 12.7 ^a^	0.002
	Aspartic Acid	34.3 ± 5.6	25.7 ± 1.3	31.2 ± 7.6	36.6 ± 4.2	0.135
	Glutamic Acid	96.1 ± 6.8	82.7 ± 3.1	88.1 ± 24.1	99.3 ± 6.4	0.437
	Glycine	32.6 ± 1.4	28.8 ± 1.2	29.1 ± 7.7	35.9 ± 0.6	0.169
	Serine	40.3 ± 14.2 ^ab^	27.1 ± 1.1 ^b^	56.5 ± 15.4 ^a^	66.1 ± 3.3 ^a^	0.009

* *p*-value represents statistical result from one-way ANOVA analysis, with post hoc multiple comparisons indicated within the Table. Means not sharing superscript letters within a row indicate statistical significance (*p* < 0.05). ^1^ Peaks for the amino acid proline were evident in some samples, but its presence was below the limit of quantification.

**Table 3:** Acid coagulation properties of infant formula (IF) samples treated with 2% (*w*/*v*) GDL at 30 °C. Data presented as mean of three independent replicates ± standard deviation. B-IF (bovine IF), C-IF (caprine IF), S-IF (soy IF), and R-IF (rice IF).

Infant Formula	Gel Point (s)	Gel pH	G’ at pH 4.6 (Pa)	G* at pH 4.6 (Pa)
B-IF	760 ± 35 ^a^	5.18 ± 0.05 ^a^	3.56 ± 0.11 ^a^	3.71 ± 0.12 ^a^
C-IF	1270 ± 35 ^b^	5.10 ± 0.02 ^a^	6.57 ± 0.22 ^b^	6.89 ± 0.23 ^b^
S-IF	N/A	N/A	0.368 ± 0.06 ^c^	0.393 ± 0.07 ^c^
R-IF	N/A	N/A	0.118 ± 0.03 ^c^	0.406 ± 0.01 ^c^

* N/A = Not applicable. Different superscript letters within a column indicate statistical significance (*p* < 0.05).

## Data Availability

The data presented in this study are available on re-quest from the corresponding author.

## Supplementary Materials

The following supporting information can be downloaded at: https://www.mdpi.com/article/10.3390/foods12071469/s1, Table S1: Chromatographic conditions for reverse phase HPLC analysis. Figure S1: PCA scores plot based on FAA analysis of intestinal digestates of infant milk formulae (IF). Figure S2: Concentration of free amine groups (mmol/g digested protein) in reconstituted (undigested) and digested infant formulae (IF) at various gastric (G0, G30, G60) and intestinal (I60) time points grouped by sample type. B-IF (bovine IF), C-IF (caprine IF), S-IMF (soy IF), R-IF (rice IF).
