# Supplementary material for: The Protein Composition and In Vitro Digestive Characteristics of Animal- versus Plant-Based Infant Nutritional Products
Source: Foods. 2023 Mar 30;12(7):1469. doi: 10.3390/foods12071469 (PMC10094249; doi:10.3390/foods12071469)
Supplement: Supplementary file 1 [file foods-12-01469-s001.zip › foods-2236269-supplementary.pdf]

Supplementary Table & Figures

Table S1. Chromatographic conditions for reverse phase HPLC analysis.

Time (min)	*Eluent B (%)*	*Conditions*
0	*5*	*initial*
0-20	*5*	*isocratic*
21-60	*50*	*linear*
61-66	*95*	*linear*
67-73	*95*	*isocratic*

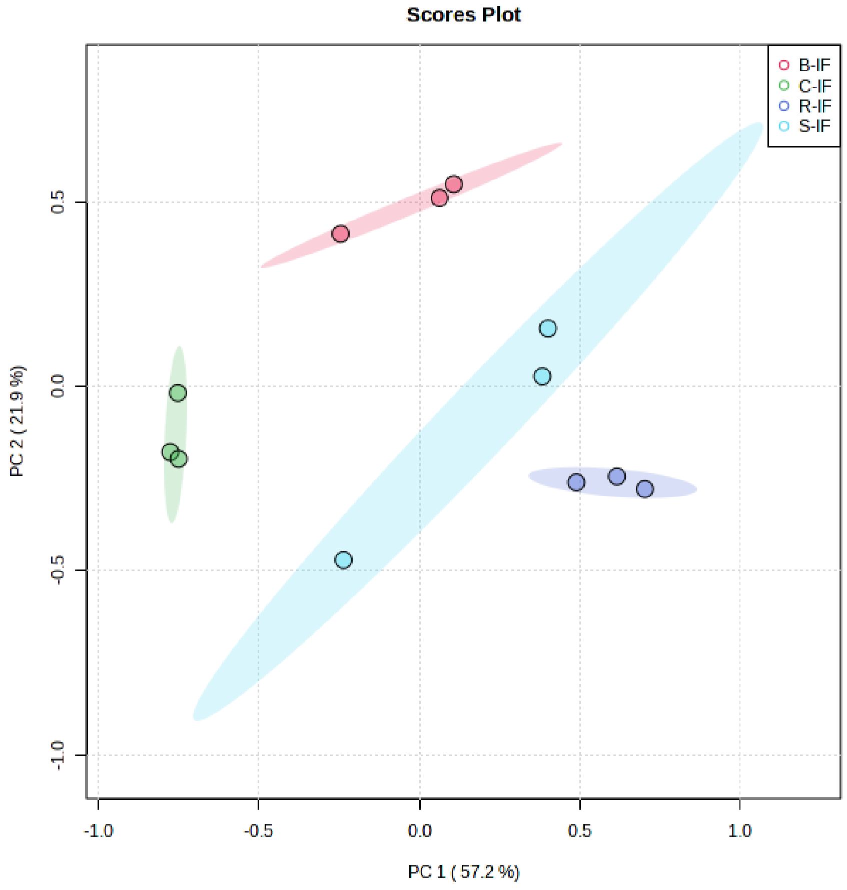

Figure S1. PCA scores plot based on FAA analysis of intestinal digestates of infant milk formulae (IF). Samples manufactured from the same protein source share the same colour. B-IF (bovine IF), C-IF (caprine IF), R-IF (rice IF), S-IF (soy IF).

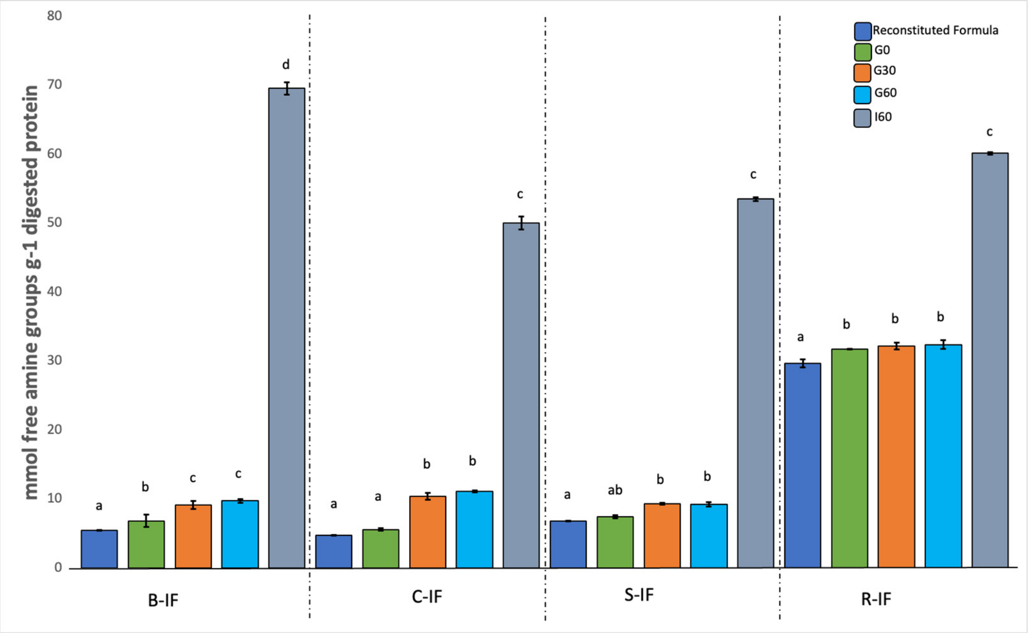

Figure S2. Concentration of free amine groups (mmol/g digested protein) in reconstituted (undigested) and digested infant formulae (IF) at various gastric (G0, G30, G60) and intestinal (I60) time points grouped by sample type. B-IF (bovine IF), C-IF (caprine IF), S-IMF (soy IF), R-IF (rice IF). Data are given as mean of three independent replicates ± standard deviation. Means not sharing superscript letters within a sample type represents statistical significance ($p < 0.05$).
